# Autonomous Cargo Transport with Biohybrid Microswimmers Enabled by Light‐Mediated Bacteria‐Cargo Communication

**DOI:** 10.1002/adma.72950

**Published:** 2026-03-30

**Authors:** Xiaoran Zheng, Yanjun Zheng, Ali Heidari, Seraphine V. Wegner

**Affiliations:** ^1^ Institute of Physiological Chemistry and Pathobiochemistry University of Münster Münster Germany

**Keywords:** autonomous cargo delivery, BcLOV4, biohybrids, light‐mediated communication, microrobots

## Abstract

Biohybrid microswimmers, which integrate the unique mobility and taxis of living cells with the versatility of synthetic cargo, offer exciting opportunities for targeted delivery. However, current biohybrids lack autonomous decision‐making capabilities due to the absence of communication between living and synthetic components. Here, we report biohybrid microswimmers capable of self‐regulating cargo pickup, transport, and release through light‐mediated communication between bacteria and cargo. The genetically engineered *Escherichia coli* bacteria act as senders, converting dynamic changes in the concentration of a model toxin, Hg^2+^, into a cellular light signal. The cargo, composed of small unilamellar vesicles (SUVs), is functionalized with a photoswitchable membrane‐binding protein to perceive the light signal. By interfacing the two components, the bacteria can dynamically signal the presence of Hg^2+^ to the SUVs, triggering their attachment to bacteria and biohybrid assembly. The inherent negative chemotaxis of bacteria to Hg^2+^ directs the transport of cargo toward low Hg^2+^ environments, where the cessation of light signaling prompts cargo release. This autonomous cargo transport is governed by an emerging self‐regulatory network, combining light‐mediated communication between cargo and bacteria with bacterial chemotaxis. The modular biohybrid microswimmer design paves the way for advanced microrobotic systems in which synthetic and living components coordinate their actions.

## 1 Introduction

Biohybrid microrobots, which combine motile living cells with synthetic cargo, have emerged as a transformative technology for drug delivery [1], biosensing [2], and environmental remediation [3]. In these systems, on the one hand, microorganisms such as bacteria and algae bring to the table capabilities such as self‐propulsion, navigation through complex biological terrain, tactic motility in response to various stimuli [4, 5, 6], and the potential for genetic engineering [7]. On the other hand, synthetic cargo can be designed to carry highly effective therapeutics, introduce new catalytic activity, and enhance the microrobot's functionality [8, 9, 10]. The action of biohybrid microrobots has been regulated by using natural tactic responses to biochemical gradients [11], applied external magnetic fields [12], and external stimuli such as pH [13] and light [14], enabling on‐demand cargo release. Although these approaches allow for supervised operation, current biohybrids lack the ability to self‐regulate their action and make decisions on the execution of complex tasks autonomously in response to their environment [15].

In most biohybrid designs, living and synthetic components function additively: microorganisms primarily serve as cargo carriers, while the cargo is viewed as a therapeutic agent to be delivered. Yet, there is no communication between these two components, preventing coordinated actions and limiting the biohybrid's ability to adapt to environmental changes. In contrast, intercellular communication in multicellular systems [16] such as tissues and biofilms gives rise to highly complex and diverse behaviors—an ability still lacking in hybrid microrobots. At the same time, microorganisms possess remarkable sensing capabilities that could be harnessed to relay information about the environment to the cargo. On the path to communication in biohybrid microrobots, synthetic cell‐like compartments capable of chemical communication with living cells have been developed [17, 18, 19]. However, engineering chemical communication between bacteria and cargo within biohybrid robots is not straightforward, due to the bacteria's intricate native biochemical signaling, which is not matched by the cargo's limited functional complexity and chemical‐loading capacity. In addition, chemical signals locally released from the senders rapidly dilute in the surrounding environment if not sustained, making it difficult for the receivers to detect them and respond, especially given the microrobot's mobility.

**FIGURE 1:**
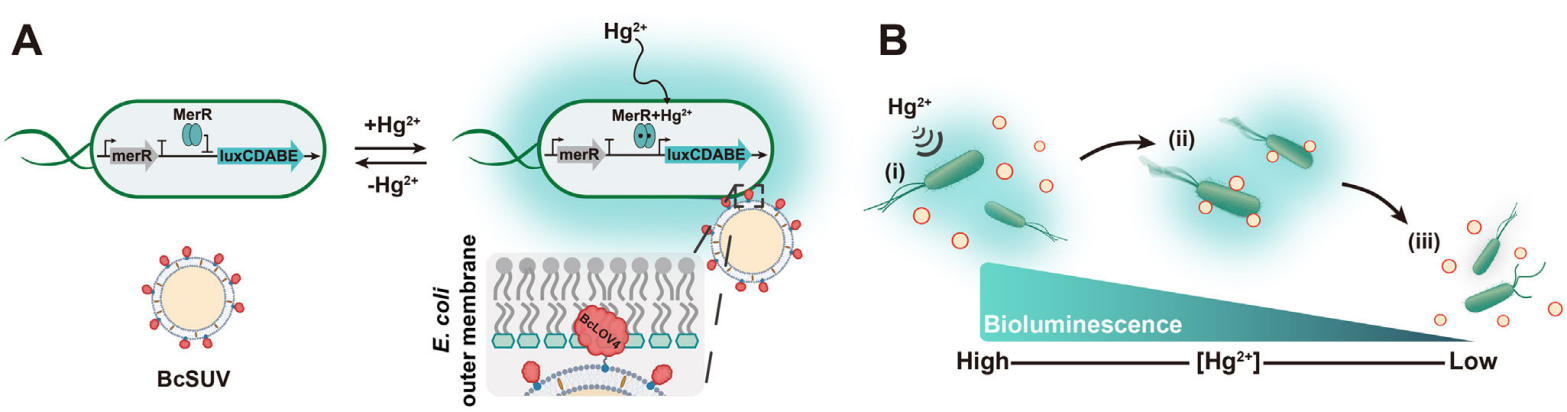
Design of bacterial biohybrid microswimmer for autonomous cargo delivery. (A) Schematic illustration of the dynamic assembly and disassembly of the bacterial biohybrid, regulated by light‐mediated communication between engineered *E. coli merR‐lux* bacteria and the cargo; SUVs functionalized with the blue light‐responsive protein BcLOV4 (BcSUVs). In the presence of Hg^2+^, the *merR‐lux* bacteria produce a bioluminescent signal, which is detected by BcSUVs. Consequently, BcSUVs bind to the bacteria, leading to the assembly of bacterial biohybrids. Conversely, in the absence of Hg^2+^, the bacteria stop luminescence production, causing the unbinding of the BcSUV cargo. (B) An overview of autonomous cargo transport in the Hg^2+^ gradient. (i) Under high Hg^2+^ conditions, *merR‐lux* bacteria signal the presence of the toxin to BcSUVs via a bioluminescence light signal, triggering the formation of biohybrid microswimmers. (ii) Bacteria transport the cargo toward lower Hg^2+^ concentrations due to innate negative chemotaxis to Hg^2+^. (iii) Under low Hg^2+^ conditions, bacteria stop producing bioluminescence, resulting in cargo release.

Here, we have engineered a nonchemical communication pathway between bacteria acting as senders and cargo acting as receivers, using light as the intercellular signal. This new‐to‐biology light‐mediated communication design allows us to create biohybrid microswimmers that autonomously assemble and disassemble to adapt to environmental changes. Unlike chemical signaling, this communication operates independently from existing biochemical signaling pathways, transmits the signal instantly from the sender to the receiver, does not rely on diffusion, requires no dedicated transporters, and remains unaffected by the movement of the biohybrid [20]. In our design (Figure 1), bacteria use the light signal to communicate to the cargo the presence of toxic mercury(II) (Hg^2+^) ions and instruct the cargo to bind to them. The bacteria then leverage their chemotactic abilities to transport the cargo away from the toxic environment. Once the biohybrid reaches an area with lower Hg^2+^ concentration, the bacterial light signal switches off, promoting cargo release. This self‐regulatory cargo loading and unloading mechanism is based on the luminescent Hg^2+^ reporter in the bacteria, which generates the light signal, and an optogenetic protein in the cargo, which acts as the photoreceptor. By developing clear design principles with established synthetic biology modules, we provide a blueprint for engineering autonomous biohybrids in which living and nonliving components coordinate their actions through light‐based communication.

## 2 Results

### 2.1 Light‐Regulated Binding of BcLOV4 to Bacterial Membranes

**FIGURE 2:**
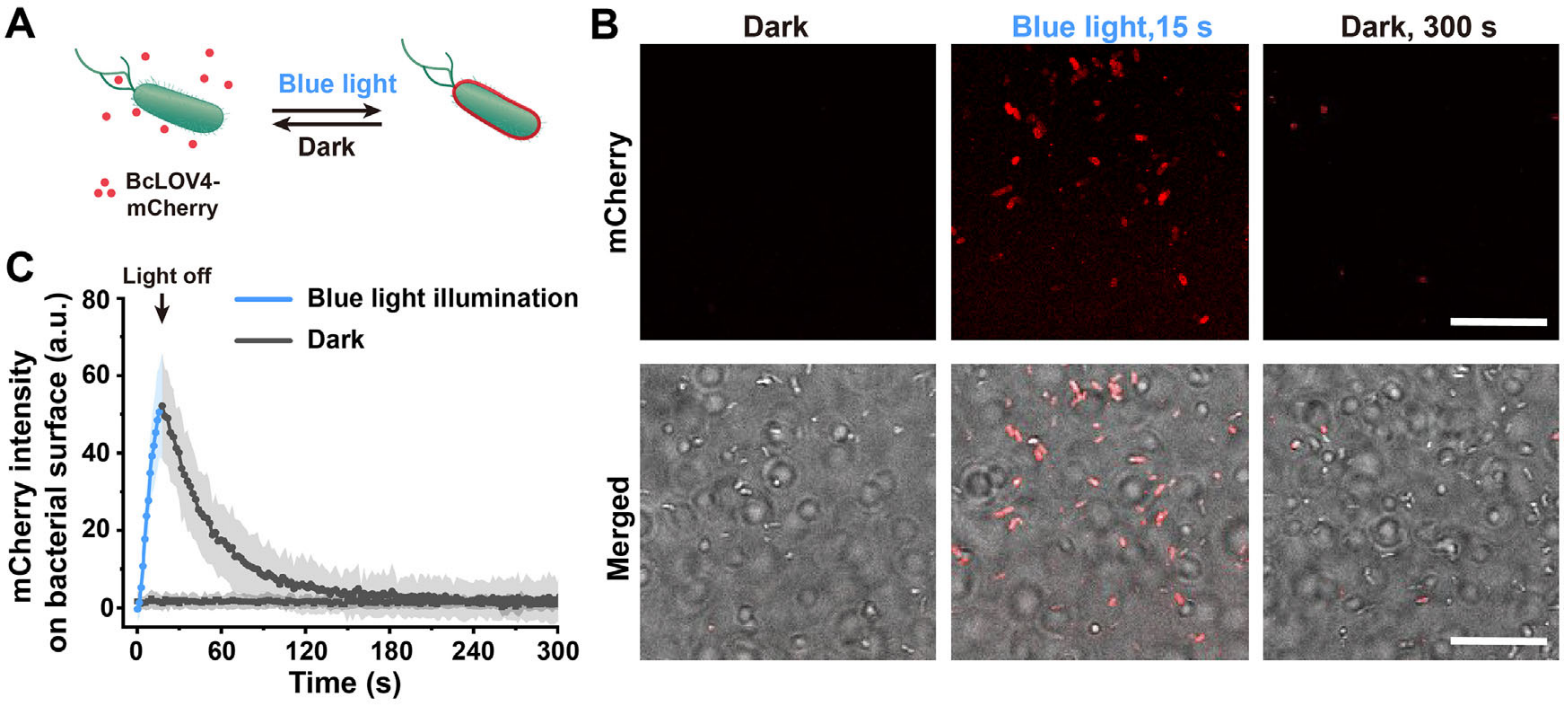
Light‐regulated membrane binding of BcLOV4 to bacterial surface. (A) Schematic showing the blue light‐triggered binding of BcLOV4 to bacteria and the reversible release in the dark. (B) Fluorescence and bright‐field microscopy images of bacterial cells in the presence of BcLOV4‐mCherry (red), first exposed to blue light for 15 s and then kept in the dark for 300 s. Scale bars are 20 µm. (C) The kinetics of light‐dependent BcLOV4‐mCherry binding to the surface of bacterial cells. The data represent the average across four independent experiments with at least 50 bacterial cells analyzed per experiment.

To achieve light‐mediated communication in biohybrid microswimmers, we searched for a protein that could bind to the bacterial outer membrane on light illumination without requiring modifications to the bacterial surface. Given the overall negative charge of the outer bacterial membrane due to negatively charged lipids and lipopolysaccharides, we hypothesized that the blue light‐switchable protein BcLOV4 could fulfil this role (Figure 2A). BcLOV4 from *Botrytis cinerea* has previously been reported to associate with negatively charged phospholipid membranes, including synthetic lipid vesicles and the inner plasma membrane of mammalian cells, due to a positively charged amphipathic region that is exposed on light activation [21]. To test this hypothesis, we incubated *Escherichia coli* (*E. coli)* MG1655 cells with purified BcLOV4‐mCherry protein (shown in red) and visualized the interaction by using bright field and fluorescence microscopy (Figure 2B). In the dark, BcLOV4‐mCherry did not bind to the bacterial membrane. However, within 15 s of blue light illumination, it rapidly associated with the bacteria. Once the blue light illumination was stopped, the mCherry fluorescence signal on the bacterial surface gradually diminished, returning to background levels within about 1 min (Figure 2C). These results reveal a previously unreported activity of BcLOV4: its binding to bacterial membranes on blue light illumination and reversible dissociation from the bacterial membrane in the dark.

### 2.2 Exploiting Cellular Bioluminescence for Protein Recruitment to Bacteria

**FIGURE 3:**
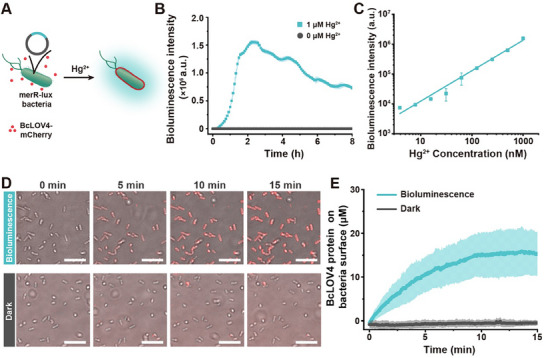
BcLOV4‐mCherry recruitment induced by bacterial bioluminescence. (A) Schematic showing the bioluminescence‐triggered binding of BcLOV4‐mCherry to *merR‐lux* bacteria, which produce bioluminescence in the presence of Hg^2+^ ions. (B) Bioluminescence signal from *merR‐lux* bacteria. (C) Bioluminescence signal from *merR‐lux* bacteria 3 h after exposure to different concentrations of Hg^2+^. (D) Overlaid microscopy images of bioluminescence‐producing *merR‐lux* bacteria (bright field, gray) show increased BcLOV4‐mCherry (fluorescence, red) recruitment over time but not for non‐luminescent bacteria (dark). Scale bars are 10 µm. (E) The kinetics of light‐dependent BcLOV4‐mCherry binding to bacteria. The data represent the average across three independent experiments with at least 50 bacteria analyzed per experiment.

Next, we engineered bacteria to detect the presence of the toxic metal ion Hg^2+^ and communicate this information to the cargo through a light signal. To achieve this, we introduced the bioluminescent Hg^2+^ reporter plasmid pSB403‐*merR*‐*lux* into the bacteria, enabling them to produce bioluminescence as a function of Hg^2+^ concentration (Figure 3A). This plasmid encodes the MerR transcriptional activator, which, in the presence of Hg^2+^, induces the expression of the downstream bacterial luciferase gene cluster (i.e., *luxCDABE*) [22]. We showed that *E. coli* MG1655 transformed with the pSB403‐*merR‐lux* plasmid (denoted as *merR‐lux* bacteria) produces strong bioluminescence for over 8 h in the presence of 1 µm Hg^2+^, with no background photon emission in the absence of Hg^2+^ (Figure 3B). Furthermore, the bioluminescence intensity of *merR‐lux* bacteria exhibited a linear correlation with Hg^2+^ concentration across a broad range (Figure 3C).

Then, we explored whether the bioluminescence from *merR‐lux* bacteria could photoactivate BcLOV4‐mCherry binding to the bacterial outer membrane. Notably, the bioluminescence emitted by *merR‐lux* bacteria has an emission maximum at 482 nm, overlapping with the absorption spectrum of the blue light‐responsive protein BcLOV4 in the range of 450–480 nm (Figure S1). When we incubated BcLOV4‐mCherry with *merR‐lux* bacteria pretreated with Hg^2+^ to induce bioluminescence, confocal microscopy revealed a gradual increase in mCherry fluorescence on the bacterial surface over 10 min (Figure 3D). This signal intensified over time, whereas no BcLOV4‐mCherry recruitment was observed in the absence of Hg^2+^‐induced bioluminescence. Notably, as shown in Figure 3E, the binding kinetics of BcLOV4‐mCherry recruitment to the bioluminescent bacteria display saturation after about 10 min. Quantitative analysis based on an mCherry standardization curve (Figure S2) revealed that at saturation, around 15 µm of BcLOV4–mCherry accumulates locally on the bacterial surface. Collectively, these results demonstrated that the bioluminescence produced by *merR‐lux* bacteria is sufficient to photoactivate the blue light‐switchable protein BcLOV4 in solution, establishing the foundation for light‐based communication between bacteria and cargo.

### 2.3 Light‐Regulated Cargo Integration and Motility of BcSUV‐Bacterial Microswimmers

**FIGURE 4:**
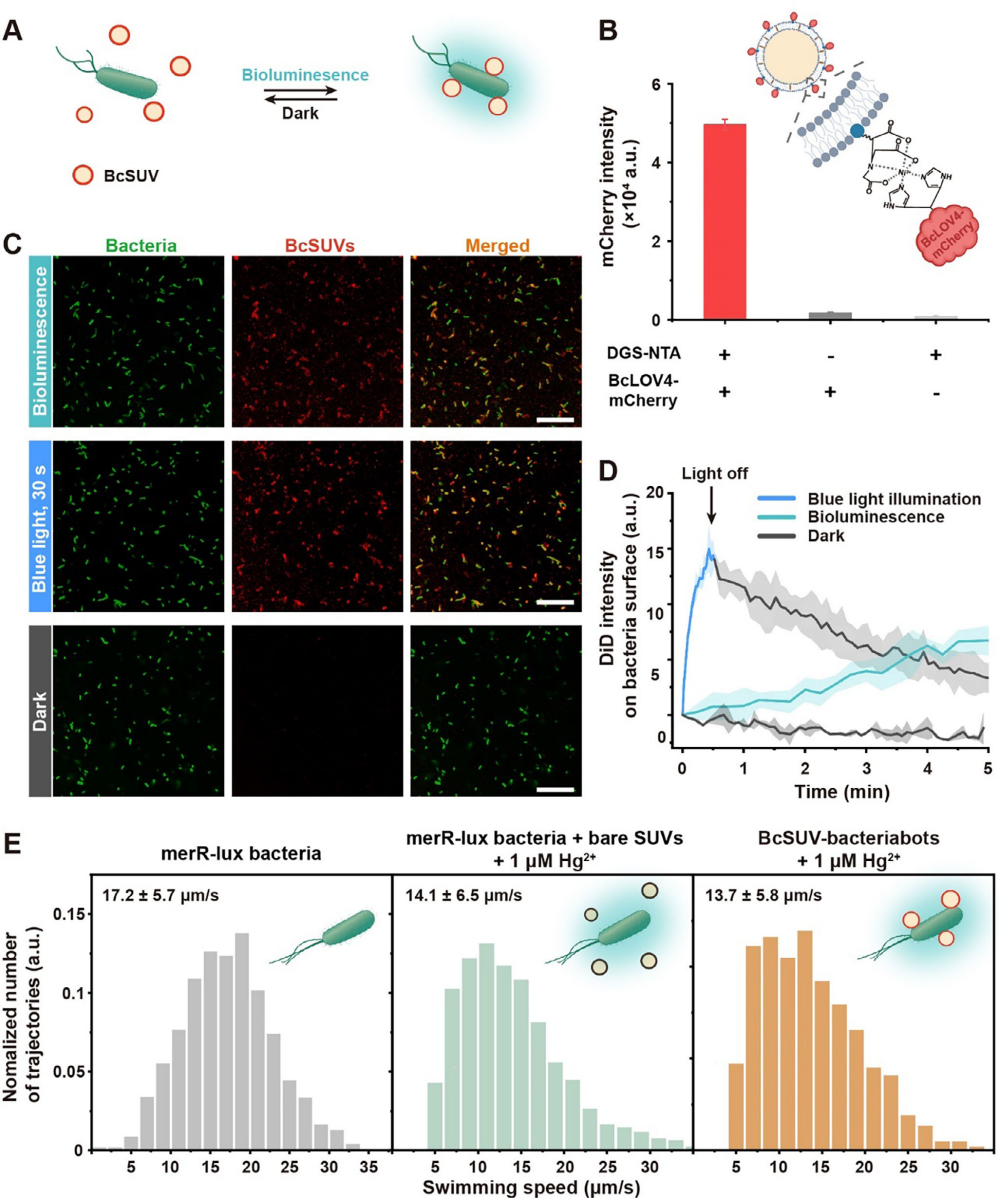
Light‐regulated cargo integration in biohybrid microswimmers. (A) Schematic illustration of BcSUVs binding to *merR‐lux* bacteria under bioluminescence and reversible unbinding in the dark. (B) BcLOV4‐mCherry functionalization of SUVs containing DGS‐NTA lipid as determined by mCherry fluorescence on the SUVs. (C) Confocal laser scanning microscopy (CLSM) images of BcSUVs (DiD channel, shown in red) interacting with *merR‐lux* bacteria (co‐expressing mCherry, shown in green) under bioluminescence (5 min), blue light (30 s), and in the dark (5 min). Scale bars are 20 µm. (D) Assembly and disassembly kinetics of BcSUV‐bacterial biohybrids were analyzed from the samples in (C). Blue light illumination (blue line) applied for 30 s and not afterward (gray line). Results represent the average across three independent experiments with at least 50 bacteria analyzed per experiment. Shaded areas are the standard deviation of the mean. (E) swimming speed distributions of *merR‐lux* bacteria, *merR‐lux* bacteria + bare SUVs + 1 µm Hg^2+^, and BcSUV‐bacteriabots + 1 µm Hg^2+^, respectively. At least 1000 trajectories were analyzed for each independent experiment (*n* = 2).

To achieve the envisioned light‐mediated communication between bacteria and cargo, we designed small unilamellar vesicles (SUVs) functionalized with BcLOV4‐mCherry as the photosensory element (Figure 4A). SUVs (DOPC: DGS‐NTA‐Ni^2+^: cholesterol: DiD molar ratio 64: 0.9: 35: 0.1) were formed by using lipid extrusion, with an average diameter of 160 nm, as determined by dynamic light scattering (Figure S3). Subsequently, BcLOV4‐mCherry protein was immobilized on the SUV surface (referred to as BcSUV hereafter) via interactions between His6‐tags on the BcLOV4‐mCherry protein and DGS‐NTA‐Ni^2+^ lipids in the SUVs (Figure 4B). Successful functionalization of the SUVs was confirmed by a strong mCherry fluorescence emission of the BcSUV solution after removing excess protein with dialysis, whereas control samples of SUVs lacking DGS‐NTA‐Ni^2+^ lipids, or SUVs not incubated with BcLOV4‐mCherry, showed no fluorescence (Figure 4B). At the same time, functionalization with BcLOV4‐mCherry did not noticeably alter the SUV size (Figure S3). Importantly, BcSUVs were also stable in different buffers over 7 days, further demonstrating their potential use in different settings (Figure S4).

Next, we combined the BcSUVs (cargo) with bioluminescent bacteria to determine whether light‐mediated communication takes place in the biohybrid microrobots. For better visualization, we co‐transformed *E. coli* MG1655 pSB403‐*merR‐lux* (*merR‐lux* bacteria) with the pTrc‐mCherry plasmid (Figure 4C). Using confocal microscopy, we observed that BcSUVs (labeled with DiD fluorophore in the membrane, λex = 638 nm, λem = 640–750 nm, shown in red) bound to the surface of bioluminescent *merR‐lux‐mCherry* bacteria (shown in green, λex = 552 nm, λem = 580–635 nm; BcLOV4‐mCherry fluorescence on the BcSUVs is much weaker and not observed on BcSUVs) (Figure 4C). In control experiments in the absence of Hg^2+^‐induced bioluminescence, BcSUVs localized to the bacterial surface on blue light illumination, whereas no BcSUVs recruitment was observed in the dark. Similar results were also obtained with *merR‐lux* bacteria not expressing mCherry (Figure S5). Negative control experiments with non‐functionalized or only GFP‐functionalized SUVs demonstrated that these SUVs lacking BcLOV4 do not bind unspecifically to bioluminescent *merR‐lux* bacteria (Figure S6). More importantly, BcSUVs are only bound to bioluminescent *merR‐lux* bacteria even in the presence of non‐luminescent mCherry‐labeled bacteria (Figure S7). These results show that bioluminescence produced by *merR‐lux* bacteria does not lead to BcSUVs accumulation on adjacent non‐luminescent bacteria and thereby, the BcSUV loading to bioluminescent bacteria is a highly specific process to control the behaviors of microswimmers. Interestingly, BcSUV binding kinetics to the bacteria differed between bioluminescence‐induced activation and external light illumination (Figure 4D). Although BcSUVs gradually bound to bioluminescent bacteria over 5 min, they rapidly attached within 30 s when exposed to external blue light. Notably, BcSUVs dissociated from the bacteria once blue light illumination was stopped, demonstrating the reversibility of BcLOV4‐mediated cargo binding to the bacteria.

Bacterial motility is a crucial factor in the development of bacterial microswimmers, as their self‐propulsion must remain unimpaired. Therefore, we analyzed the swimming trajectories and average swimming speed of the *merR‐lux* bacteria in the presence and absence of BcSUVs and 1 µm Hg^2+^ (added to induce bioluminescence). First, the transformation of *E. coli* MG1655 with the pSB403‐*merR‐lux* plasmid to generate *merR‐lux* bacteria had no effect on its motility (Figure 4E; Figure S8). For *merR‐lux* bacteria, the presence of Hg^2+^ and bare SUVs reduced the average speed from 17.2 ± 5.7 to 14.1 ± 6.5 µm s^−^^1^ (Figure 4E; Figure S8 *merR‐lux* bacteria in the presence of Hg^2+^ without SUVs 16.2 ± 5.7 µm s^−^^1^). Although Hg^2+^ exposure led to a 15% reduction in swimming speed, the bacteria remained highly motile. Furthermore, when both Hg^2+^ and BcSUVs were present, the average speed remained similarly high at 13.7 ± 5.8 µm s^−1^. These results indicated that neither BcSUV loading onto the bacteria nor Hg^2^^+^ exposure significantly impaired bacterial motility. In addition, we show that 1 µm Hg^2+^ did not impair bacterial growth and Hg^2^^+^ only started toinhibit growth at concentrations above 3 µm (Figure S9). These results indicate that at 1 µm Hg^2+^, the bacterial microswimmers are responding to relevant concentrations of Hg^2+^ close to the toxicity so that the microswimmers can carry the cargo away from it.

### 2.4 Adaptive Cargo Integration Regulated by Environmental Signal

**FIGURE 5:**
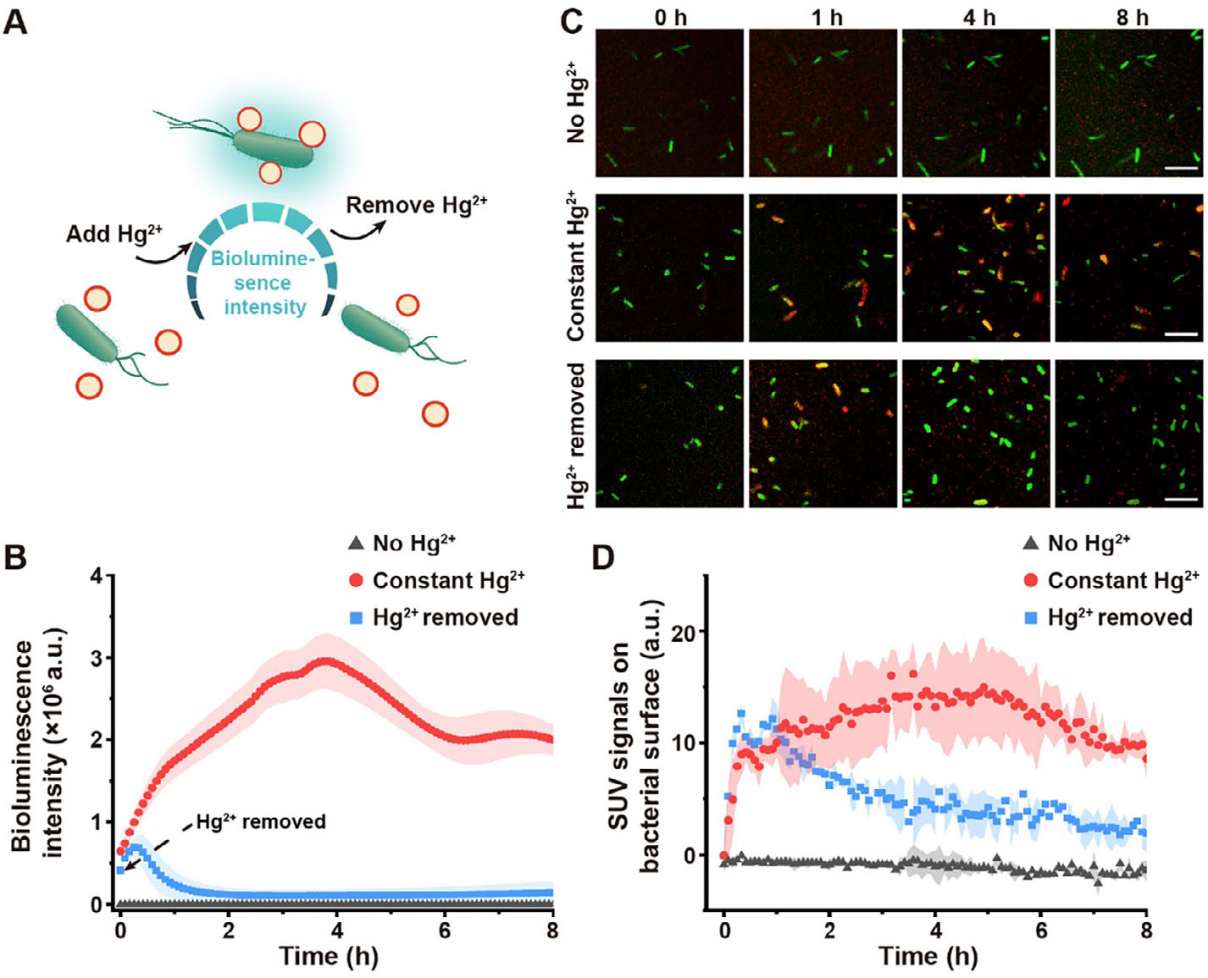
Adaptive cargo integration into bacterial microswimmers regulated by environmental Hg^2+^ abundance (A) Schematic of self‐regulated cargo binding and release depending on Hg^2+^ availability. (B) Bioluminescence intensity in response to changing Hg^2+^ in the environment. The *merR‐lux* bacteria were preincubated with no or 1 µm Hg^2^^+^ for 3 h, before Hg^2+^ was removed or kept constant. (C) CLSM images of BcSUVs (DiD channel, shown in red) interacting with *merR‐lux* bacteria (co‐expressing mCherry, shown in green) that were exposed to no Hg^2+^, constant Hg^2+^, or removal of Hg^2+^. Scale bars are 10 µm. (D) Kinetic analysis of BcSUV binding to *merR‐lux* bacteria in (C), showing the dynamic assembly and disassembly of BcSUV‐bacterial biohybrids in response to changing Hg^2+^ abundance. Results represent the average across three independent experiments with at least ten bacteria analyzed per experiment.

A key requirement for autonomous bacterial microswimmers is their ability to reversibly assemble and disassemble in response to changes in the environment. Having established that microswimmer assembly is driven by light‐based communication between *merR‐lux* bacteria and BcSUV cargo, we explored whether this process was reversible when the light signal from the sender diminished (Figure 5A). We first examined how the cellular light output changes in response to decreasing environmental Hg^2+^ levels, as part of the bacterial microswimmers’ self‐regulation mechanism. When *merR‐lux* bacteria were first exposed to 1 µm Hg^2^^+^ for 3 h, they produced significant bioluminescence. However, on removal of Hg^2+^ through a washing step, bioluminescence rapidly declined within 1 h (blue line, Figure 5B). In contrast, bacteria that were re‐supplemented with Hg^2+^ after the removal step continued to produce high bioluminescence over 8 h (red line, Figure 5B). The decrease in bioluminescence intensity following the removal of Hg^2+^ is presumably results from the degradation of the *luxCDABE* gene products catalyzing the bioluminescence reaction. On the other hand, the presence of Hg^2+^ ensures continuous expression of these genes, thereby sustaining bacterial luminescence.

Next, we explored how dynamic changes in the light signal from the sender impact the kinetics of cargo attachment/detachment. Right after Hg^2+^ was removed from the medium containing *merR‐lux* bacteria, BcSUVs added to the bacteria still rapidly recruited to their surfaces because of the strong bioluminescence within the first hour (Figure 5C; Movies S1–S3). Similarly, BcSUVs were bound to bacterial cells under constant exposure to Hg^2^^+^ ions. However, although both groups exhibited cargo attachment in the first hour, BcSUVs later detached from bacteria where Hg^2^^+^ had been removed, leading to transient assembly of BcSUV bacterial microswimmers in response to environmental Hg^2^^+^ availability (Figure 5D). In contrast, cargo remained stably attached to the bacteria maintained under constant Hg^2^^+^ levels, and no cargo attachment was observed in the sample that had never been exposed to Hg^2+^. Together, these findings demonstrate that light‐based communication between bacteria and cargo enables the dynamic assembly and disassembly of bacterial microswimmers in response to environmental chemical signals due to the implemented gene regulatory network in the bacteria.

### 2.5 Autonomous Cargo Transport by BcSUV‐Bacterial Biohybrid Microswimmers

**FIGURE 6:**
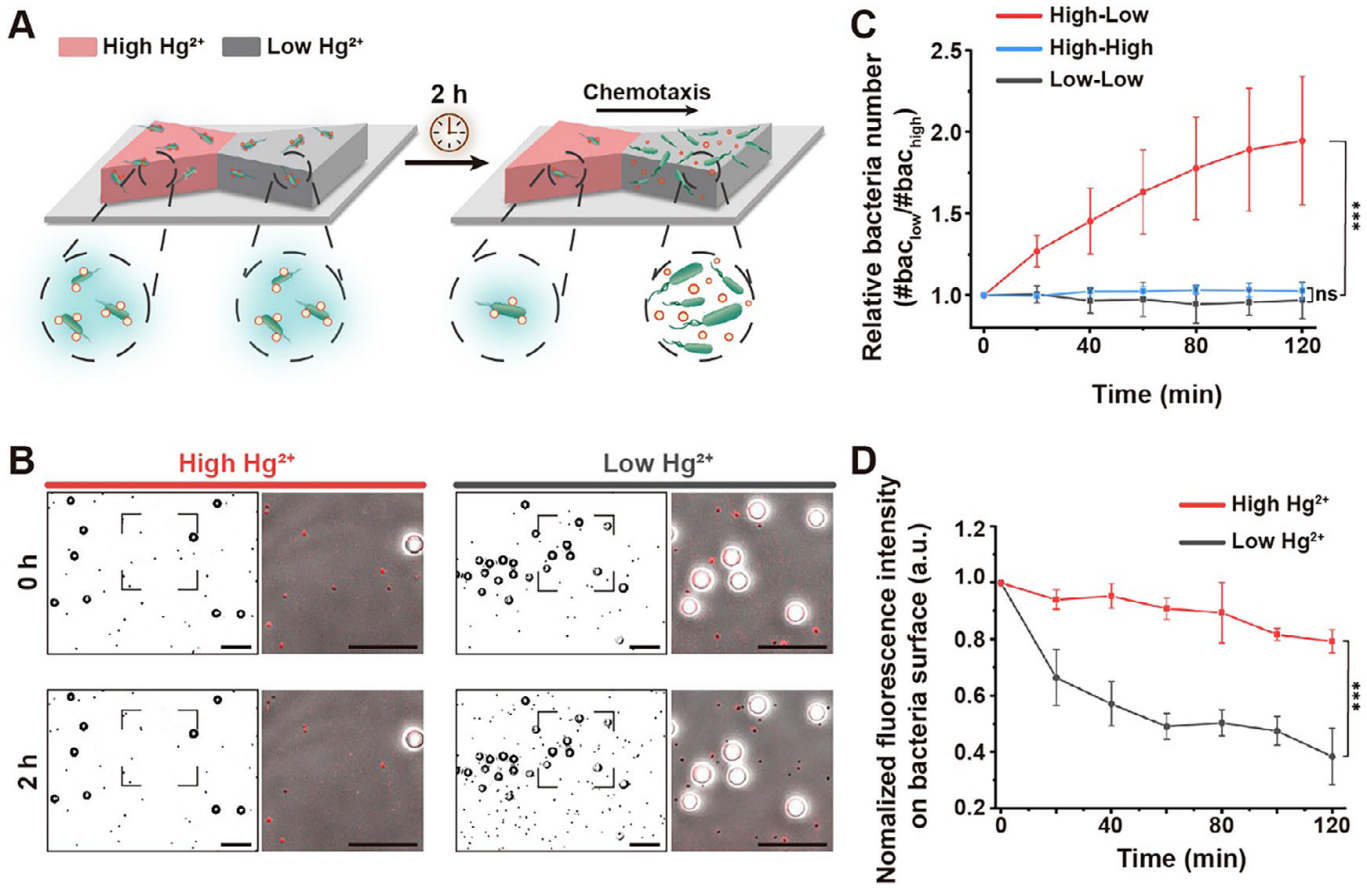
Autonomous cargo delivery in a chemotactic gradient. (A) Schematic representation of preassembled BcSUV‐*merR‐lux* bacterial microswimmers loaded in a chemotaxis chamber with two compartments filled with high (1 µm) and low (0 µm) Hg^2+^ concentrations. The initially evenly distributed bacterial microswimmers move from the high Hg^2+^ to the low Hg^2+^ side and, once there, drop the BcSUV cargo due to the *merR‐lux* bacteria's lowered bioluminescence under these conditions. (B) The *merR‐lux* bacteria (shown as black dots on a white background in a binary image generated from a phase contrast image) were initially evenly distributed in both the high and low Hg^2+^ sides. 12 µm PS beads (black‐rimmed hollow circles) were added as obstacles. BcSUV fluorescence (shown in red) overlaid with the phase contrast images of bacteria (zoomed in area outlined by dashed lines in the binary image). After 2 h, the number of bacterial cells was higher in the low Hg^2+^ than in the high Hg^2+^ side, and no BcSUV fluorescence was observed on the bacterial surface in the low Hg^2+^ side, indicating the unbinding of the cargo. Scale bars are 50 µm. (C) Quantification of the relative number of bacterial cells in the low and high Hg^2+^ compartments in a field of view of 700 µm × 700 µm. At t = 0 min there were at least 500 bacteria per field of view and the results are presented as the average of three independent experiments. (D) BcSUVs signal on the bacterial surface in the high and low Hg^2+^ compartments of the chemotaxis chamber for the experiment presented in (B). The fluorescence intensity at different time points was normalized against the average fluorescence intensity at 0 min. Data were acquired from three independent experiments (*n* = 3) with > 100 bacteria analyzed per experiment. ^***^ *p* < 0.001, ns: not significant.

Biohybrid microrobots capable of performing autonomous tasks without human supervision are an exciting and promising concept [23]. Toward this goal, we propose using the bacterial microrobots developed here, leveraging their intrinsic ability to sense and move away from toxic molecules such as Hg^2+^. This design – combining adaptive cargo loading with the bacteria's negative chemotaxis—enables bacteria to pick up cargo in high Hg^2+^ regions and transport it to the areas with lower Hg^2+^ concentrations, where they subsequently release the cargo (Figure 6A). In an initial experiment, we introduced mCherry‐labeled bacteria into a chemotaxis chamber, where one side contained a high concentration of Hg^2+^ (1 µm) and the other a low concentration of Hg^2+^ (0 µm). Here, we observed that the bacteria moved to the low Hg^2+^ chamber within 120 min (Figure S10). As a proof‐of‐concept for the autonomous delivery, we first preassembled bacterial microswimmers from *merR‐lux* bacteria and BcSUVs in the presence of Hg^2+^ and introduced them into a chemotaxis chamber as described above. Moreover, we included 12 µm polystyrene (PS) beads as obstacles for the bacteria, more reminiscent of environmental conditions that bacteria would need to navigate. Over 2 h, we observed that the bacteria (shown in black on a white background, PS beads as hollow back circles) migrated toward the low Hg^2^^+^ side (Figure 6B). Simultaneously, the fluorescence signal from BcSUVs on the bacteria's surface (shown in red) decreased on the low Hg^2+^ side, indicating cargo release. To quantify these observations, we monitored bacterial distribution and BcSUV fluorescence on the bacteria in both the high and low Hg^2+^ sides of the chemotactic chip over time (Figure 6C,D). Initially, bacteria were evenly distributed between the two chambers. However, after 2 h, the number of bacteria in the low Hg^2+^ side was twice as high as on the high Hg^2+^ side (Figure 6C). At the same time, BcSUV fluorescence on the bacterial surface decreased by 51% within 1 h in the low Hg^2^^+^ region, whereas in the high Hg^2^^+^ region, the decrease was only 9% (Figure 6D), showing a significant difference in BcSUV signals on the bacterial surface between the two regions. In these experiments, the presence of the PS beads had no effect, demonstrating the ability of the bacterial microswimmers to navigate obstacles. Meanwhile, in two control experiments with equally high or low Hg^2^^+^ concentration on both sides showed an even distribution of bacteria in the two chambers (Figure 6C). Moreover, in these control experiments, we observed that BcSUVs remain on the bacteria under high Hg^2^^+^ and dissociate from the bacteria under low Hg^2^^+^ concentrations, further confirming the cargo release is driven by environmental Hg^2+^ variation (Figure S11). Overall, these observations revealed that BcSUV‐loaded bacterial microswimmers can autonomously transport cargo by integrating bacterial motility, environmental responsiveness, and adaptive cargo integration.

## 3 Conclusion

We designed a biohybrid microswimmer capable of autonomously picking up cargo in high Hg^2+^ environments and releasing it once it reaches low Hg^2+^ conditions. This task is enabled by self‐regulatory networks arising from Hg^2+^‐dependent bacterial bioluminescence and light‐mediated communication between the living and synthetic components of the biohybrid. Specifically, genetically engineered bacteria convert environmental Hg^2+^ signals into intracellular light signals, actively communicating to the cargo, which is equipped with the photoreceptor BcLOV4. The light‐activated and reversible binding of BcLOV4‐functionalized cargo to bacteria enables dynamic and autonomous assembly and disassembly of the biohybrid in response to bacterial bioluminescence. Coupled with the bacteria's intrinsic chemotactic motility, the resulting BcSUV‐bacterial microrobots can transport and release cargo, enabling adaptive cargo navigation. One can envision that such mercury‐dependent cargo transport could be useful for bioremediation or for enabling synthetic systems to avoid toxic environments. In the chemotactic assay, we showed that the presence of microbeads had no effect on performance, suggesting that these microswimmers may be capable of navigating more complex environments. Compared with microrobotic designs that use discrete electronic components [24] or synthetic materials with geometric configurations [25, 26], the complexity in the biohybrid arises from the communication between the living and synthetic components. As a result, the BcSUV‐bacterial microswimmers are capable of executing complex autonomous tasks, eliminating the need for external human supervision.

Light‐based communication between living and synthetic components has introduced a new dimension in biohybrid interactions. Beyond examples of chemical communication between synthetic compartments and living cells, the feasibility of light‐mediated communication at the microscale has been demonstrated between synthetic sender‐receiver lipid vesicles [27], and neuronal cells [28]. In addition, luminescence produced in synthetic vesicles has been shown to influence spore formation in light‐sensitive fungi [29]. However, in none of these examples are bacteria acting as the light‐producing sender, and this mode of communication has not been engineered to regulate biohybrid behavior. Notably, light‐based communication is entirely independent of existing biochemical signaling pathways in living cells, where only a few systems have the capability of producing or responding to light. Furthermore, it is relatively simple to engineer light production and responsiveness into synthetic systems by incorporating luciferases and light‐responsive proteins. Finally, the rapid diffusion‐independent propagation of light makes it an excellent way to transmit information in dynamic systems with movement and flow.

Synthetic biology allows us to precisely tailor how cells perceive, process, and translate external signals into functional outputs. Despite its vast potential, communication between biotic and abiotic interfaces remains underexplored in biohybrid microrobots [30]. For example, by modifying different modules of light‐mediated communication in this system, various adaptive and self‐regulated behaviors could be engineered. First, the Hg^2+^‐sensitive *merR‐lux* bioluminescent reporter could be replaced with a constitutively active luciferase expression or another bioluminescent reporter responsive to other environmental cues, such as oxidative stress [31], different heavy metals [32], or organic molecules [33]. These alternative bioluminescent sensors would expand the biohybrid's sensory capabilities, allowing it to respond dynamically to various environmental changes. Their potential is exemplified by the H_2_O_2_‐responsive bioluminescent reporter, in which BcSUVs recruitment was triggered by bacterial bioluminescence in response to H_2_O_2_ (Figure S12). Second, although the photoreceptor BcLOV4 in this study facilitated the adaptive assembly and disassembly of the biohybrids through photoswitchable adhesions, other photoswitchable proteins could be incorporated in the cargo to regulate alternative light‐controlled activities, such as protein synthesis [29], pore formation [34], or protein localization [35]. In fact, a number of studies indicate that luminesce can be used to photoactivate a variety of blue light‐sensitive proteins [36]. Combined with the native or engineered taxis behavior of bacteria, this design unlocks numerous possibilities for autonomous biohybrid engineering. As demonstrated in this work, the synergy between biological machinery and synthetic materials enables the creation of highly versatile self‐regulating biohybrids for applications in robotics, environmental sensing, and biomedical devices [37, 38, 39].

## 4 Materials and Methods

### 4.1 Materials

Tetracycline, isopropyl β‐D‐1‐thiogalactopyranoside (IPTG), dithiothreitol (DTT), phenylmethylsulfonyl fluoride (PMSF), imidazole, nickel(II) chloride (NiCl_2_), and mercury(II) chloride (HgCl_2_) were purchased from Sigma–Aldrich. LB medium, LB agar, and chloroform were obtained from Carl Roth. The 1000 kDa Float‐A‐Lyzer G2 Dialysis Device was acquired from Repligen. 1,2‐dioleoyl‐sn‐glycero‐3‐phosphocholine [18:1 (Δ9‐Cis) PC (DOPC)], 1,2‐dioleoyl‐sn‐glycero‐3‐[(N‐(5‐amino‐1‐carboxypentyl) iminodiacetic acid) succinyl] (nickel salt) [18:1 DGS‐NTA‐Ni^2+^], cholesterol, the mini‐extruder, and PC membranes were purchased from Avanti Polar Lipids. PureCube Ni‐NTA agarose beads (#31103) were purchased from Cube Biotech. 1,1'‐Dioctadecyl‐3,3,3',3'‐tetramethylindodicarbocyanine, 4‐chlorobenzenesulfonate salt (DiD) was obtained from Thermo Fisher Scientific. The µ‐Slide 18 Well and µ‐Slide Chemotaxis chambers were acquired from Ibidi. A genetically engineered high‐motility strain of *E. coli* MG1655 (designated as VS202) was provided by Prof. Victor Sourjik at the Max Planck Institute for Terrestrial Microbiology. The plasmid His6_BcLOV4_mCherry_BamUK (Addgene #114596; http://n2t.net/addgene:114596; RRID:Addgene_114596) was a gift from Prof. Brian Chow [21], the plasmid pSB403‐*merR‐lux* was a gift from Prof. Dr. van der Meer [40], and the plasmid pTrc‐mCherry was a gift from Prof. Victor Sourjik. The compositions of buffers used in this study are listed in Table S1 and were prepared with milli‐Q water.

### 4.2 Expression and Purification of His6‐BcLOV4‐mCherry

The plasmid His6_BcLOV4_mCherry_BamUK was transformed into *E. coli* BL21(DE3). A single colony from an agar plate was cultured overnight in 10 mL LB medium containing 50 µg mL^−1^ kanamycin at 37°C and 160 rpm. The overnight culture was then diluted 1:100 into 1 L of fresh LB medium supplemented with kanamycin and cultured at 37°C, 160 rpm until it reached an optical density at 600 nm (OD_600_) of 0.4‐0.5. Next, the protein expression was induced by adding 0.5 mm IPTG at 16°C overnight. The cells were harvested by centrifugation (6000 rpm, 12 min, 4°C). The cell pellet from 1 L culture was resuspended in 30 mL buffer A supplemented with 1 mm DTT and 1 mm PMSF. Afterward, the resuspended bacteria were lysed by ultrasonication on ice. Finally, the cell suspension was spun down at 14000 rpm for 40 min at 4°C, and the supernatant was sequentially filtered through 0.45 µm and 0.20 µm polyethersulfon syringe filters.

The His6‐BcLOV4‐mCherry was purified using a column filled with 3 mL PureCube His Affinity Agarose. Similar to the manufacturer's instructions, the column was first equilibrated with 5 column volumes (CV) of buffer A before the cell lysate was loaded at a flow rate of 2 mL min^−1^. Following this, the column was washed with 5 CV of buffer A and then with 10 CV of wash buffer. Subsequently, the protein was eluted with 3 CV elution buffer, and the fractions containing the protein were dialyzed against buffer A using a dialysis membrane with a molecular weight cut‐off of 3.5 kDa overnight at 4°C. The purified protein solution was snap‐frozen in liquid nitrogen and stored at ‐80°C for further experiments.

### 4.3 Preparation and Functionalization of Small Unilamellar vesicles (SUVs)

A 200 µL of 5 mg mL^−1^ lipid mixture in chloroform containing DOPC, 18:1 DGS‐NTA‐Ni^2+^, cholesterol, and DiD in a molar ratio of 64:0.9:35:0.1 was transferred into a 2 mL glass vial. For the control SUVs without DGS‐NTA, the addition of 18:1 DGS‐NTA(Ni) was skipped. The chloroform was evaporated using a nitrogen gas stream, and the vial was placed under high vacuum overnight to remove any residual solvent. The resulting lipid film was rehydrated with 1 mL buffer A to achieve a final lipid concentration of 1 mg mL^−1^. To form SUVs, the lipid suspension was sequentially extruded 15 times first through a 0.4 µm PC membrane, followed by a 0.1 µm PC membrane.

For the surface functionalization, 1000 µL of 1 mg mL^−1^ SUVs were mixed with 2 µm of His6‐BcLOV4‐mCherry at 4°C for 1 h. The BcLOV4‐functionalized SUVs, denoted as BcSUVs, were then transferred into a 1000 kDa dialysis device and dialyzed against the working buffer for 4 h at 4°C. The resulting BcSUVs were harvested into a 1.5 mL Protein LoBind Eppendorf tube and immediately utilized for further experiments.

### 4.4 Characterization of SUVs

To verify the successful functionalization of SUVs with His6‐BcLOV4‐mCherry, 200 µL suspensions of bare SUVs (without DiD) with or without DGS‐NTA‐Ni^2+^ were transferred into a 96‐well black plate after the dialysis step. The mCherry fluorescence intensities were measured using a plate reader (Tecan Spark, CH), with an excitation wavelength of 540 nm and an emission wavelength of 600 nm. The hydrodynamic diameters of the bare SUVs and BcSUVs was measured using dynamic light scattering (Nano ZS Zetasizer, Malvern Panalytical, UK). The stability of BcSUVs was measured in PBS buffer, working buffer, and motility buffer over 7 days using DLS.

### 4.5 Bacterial Culture

A glycerol stock of *E. coli* MG1655 (VS202) bacteria (transformed only with pSB403‐*merR‐lux* plasmid or co‐transformation with pSB403‐*merR‐lux* and pTrc‐mCherry), kept at −80°C, was freshly streaked on LB‐agar plates, and a single colony from the agar plates was cultured overnight in LB medium containing 10 µg mL^−1^ tetracycline (and 50 µg mL^−1^ ampicillin for the co‐transformed strain) under 37°C and 160 rpm. The next morning, the overnight culture was diluted 1:100 in fresh LB medium supplemented with the same antibiotics. The bacterial culture was then incubated at 37°C under shaking conditions (150 rpm) until it reached an OD_600_ of 0.4–0.5 and at harvested for further experiments (starting culture).

### 4.6 Bacterial Growth in the Presence of Hg^2+^

The starting culture of *merR‐lux* bacteria was spun down at 3000 rpm for 5 min and the bacteria were resuspended in fresh LB medium to a OD_600_ = 0.1. Then, HgCl_2_ was added to the bacteria at final Hg^2+^ concentrations up to 10 µm. Afterward, 200 µL of the bacterial suspensions with different Hg^2+^ concentrations were transferred to a 96‐well transparent plate (Greiner bio‐one, 655101) and the optical density at 600 nm was recorded every 5 min for 24 h at 37°C using a plate reader. Before each 5 min measurement interval, the bacteria were shaken for 3 s in the plate reader.

### 4.7 Bioluminescence Measurements

The starting culture of *merR‐lux* bacteria was spun down at 3000 rpm for 5 min, and the bacteria were resuspended in working buffer to an OD_600_ = 0.1. Then, HgCl_2_ was added to the bacteria suspensions at a final Hg^2+^ concentration of 0–1 µm. Afterward, 200 µL of the bacterial suspensions with different Hg^2+^ concentrations were transferred to 96‐well white plate (Greiner bio‐one, 655075) and the bioluminescence intensities between 400 and 500 nm was measured every 5 min for 24 h at 30°C. Before each 5 min measurement interval, the bacteria were shaken for 3 s in the plate reader.

### 4.8 Recruitment of BcLOV4‐mCherry to Bacteria Surface

A starting culture of VS202 bacteria transformed with the pSB403‐merR‐lux plasmid (denoted as merR‐lux bacteria) was incubated with 1 µm HgCl_2_ for 3 h at 30°C to induce the bioluminescence production. Subsequently, the bacteria were collected via centrifugation (3000 rpm, 5 min) and resuspended in fresh working buffer at the final OD_600_ of 0.1. For the dark and light control samples, no HgCl_2_ was added. For each experiment, 80 µL of the OD_600_ of 0.1 bacterial suspension was placed into an 18‐well ibidi chamber and left undisturbed for 30 min prior to imaging. Subsequently, 1 µL of 40 µm His6‐BcLOV4‐mCherry (final concentration 500 nM) was added to each well containing the bacteria. Images were acquired using a confocal laser scanning microscope (CLSM) with a 63× water objective (SP8, Leica Microsystems, DE) in both the mCherry (λex = 552 nm, λem = 580–660 nm) and bright field channels. For blue light illumination, 0.2% of the 488 nm laser was used as the light source to activate BcLOV4‐mCherry. The output of the 0.2% 488 nm laser is 0.2 mW at the focal plane. A standardization curve for the fluorescence intensity of BcLOV4‐mCherry was established by measuring the fluorescence intensity of BcLOV4‐mCherry protein in solution (0–80 µm) using the same imaging settings as in the experiment of BcLOV4‐mCherry recruitment to the bacterial surface.

### 4.9 Light‐Activated Assembly of Bacterial Biohybrids

VS202 co‐transformed with the pSB403‐*merR‐lux* and pTrc‐mCherry plasmid, prepared and treated with 1 µm HgCl_2_ as described for the BcLOV4‐mCherry recruitment. For each experiment, 80 µL of the OD_600_ of 0.1 bacterial suspension was transferred into an 18‐well ibidi dish and allowed to settle for 15 min before imaging. Subsequently, 80 µL of 1 mg mL^−1^ BcSUVs were added to each well containing the bacteria. Subsequently, images were captured using CLSM with a 63× water objective in both the mCherry (λex = 552 nm, λem = 580–635 nm) and DiD (λex = 638 nm, λem = 640–750 nm) channels. For blue light illumination, 0.2% of the 488 nm laser was used as the light source to activate BcLOV4‐mCherry. The output of the 488 nm laser we used was 0.2 mW at the focal plane.

To verify the specific binding of BcSUVs, 80 µL of non‐functionalized or GFP‐functionalized SUVs (functionalized with His6‐tagged GFP as described above for BcLOV4‐mCherry) were used instead of BcSUVs in the above experiment, and the same imaging settings were used to observe the accumulation of these SUVs on the bacterial surface. To test the specific recruitment of BcSUVs to bioluminescent merR‐lux bacteria, these bacteria were mixed with VS202 transformed with the pTrc‐mCherry plasmid at an OD_600_ of 0.1 each. Subsequently, 80 µL of the bacterial mixture was transferred into an 18‐well ibidi dish and allowed to settle for 15 min before imaging. Next, 80 µL of BcSUVs (1 mg·mL^−^^1^) were added, and images were captured using the same settings as above.

### 4.10 Swimming Speed Analysis

After the starting culture, *merR‐lux* bacteria were treated with 1 µm HgCl_2_ as described for the BcLOV4‐mCherry recruitment. Subsequently, for the assembly of bacterial biohybrids, 100 µL of the bioluminescent bacterial suspension in working buffer with 1 µm HgCl_2_ with an OD_600_ of 0.1 was mixed with either 100 µL of bare SUVs or BcSUVs and incubated on an orbital shaker at 150 rpm for 30 min at room‐temperature. Following an established protocol [41], 5 µL of the mixtures (i.e., *merR‐lux* bacteria without any HgCl_2_ treatment, *merR‐lux* bacteria + bare SUVs + 1 µm HgCl_2_, *merR‐lux* bacteria + BcSUVs + 1 µm HgCl_2_) was placed between two coverslips and sealed with vaseline. For the 2D swimming speed analysis and trajectory characterization, phase contrast videos were acquired by an inverted light microscope and CCD camera using 10× objective at 10 frames per second (DMi8, Leica Microsystems, DE). All movies were analyzed with Fiji ImageJ to track bacterial cells. A custom‐written particle‐tracking plugin was employed to detect particles using a centroid localization algorithm [41]. The particle tracking software is available from https://github.com/croelmiyn/ParticleTracking. To link trajectories, the closest particle in the subsequent frame was identified. The instantaneous velocity *v* *_i_* (*t*) (in 2D) of an object was determined by performing a linear fit on the trajectory position *r* *_i_* (*t*) within a 10‐frame‐long sliding window centered around the time point *t*.

### 4.11 BcSUV Attachment/Detachment Kinetics with Changing Hg^2+^ Concentrations

VS202 co‐transformed with the pSB403‐*merR‐lux* and pTrc‐mCherry plasmids was prepared and threated with 1 µm HgCl_2_ for 3 h at 30°C as before. Then, the bioluminescent bacteria were spun down at 3000 rpm, 5 min and resuspended in fresh working buffer without (Hg^2+^ removed) or with (Hg^2+^) 1 µm HgCl_2_. For the no Hg^2+^ group, the addition of HgCl_2_ was skipped throughout the entire experiment. 80 µL of the OD_600_ of 0.1 bacterial suspension from each group was transferred into an 18‐well ibidi chamber and allowed to settle for 15 min before imaging. Afterward, 80 µL of BcSUVs was added to each well containing the bacteria. Images were then captured every 5 min using CLSM equipped with a 63× water objective in both the mCherry and DiD channels.

### 4.12 Chemotactic Movement and Cargo Delivery of BcSUV‐*merR‐lux* Microswimmers

After starting the culture of *merR‐lux* bacteria was treated with 1 µm HgCl_2_ as before, the luminescent bacteria were resuspended in fresh working buffer to an OD_600_ = 1. The chemotaxis assay was then performed in chemotaxis chambers according to the manufacturer's instructions (µ‐Slide Chemotaxis, Cat.No:80326), ensuring that the left reservoir contained motility buffer with 1 µm HgCl_2_, while the middle channel and right reservoir contained only motility buffer.

Next, 100 µL of the bioluminescent bacterial suspension was mixed with 100 µL of BcSUVs and incubated on an orbital shaker at 150 rpm for 30 min to assemble the biohybrids. 10 µL of the resulting bacterial microswimmer suspension was diluted into 190 µL of motility buffer. Next, 10 µL of a mixture of the diluted *merR‐lux* bacteria + BcSUVs and 0.5 mg/mL 12 µm PS beads was then pipetted into the middle channel of the chemotaxis dish. Subsequently, 10 µL of liquid was removed from the middle channel to evenly distribute the biohybrids within the chemotaxis dish. The bacteria and BcSUV‐bacterial biohybrids were tracked on an inverted fluorescence microscope with a 10× objective (DMi8, Leica Microsystems, DE) in both the DiD and phase contrast channels. Every 20 min, tile scans (total area = 3500 µm × 2500 µm) were recorded in the phase contrast and DiD channels in the chemotaxis chamber, including the high Hg^2+^ area, central channel, and low Hg^2+^ area. The acquired tile scan images were then stitched into composite images by the Mosaic merge tool using the Leica software. To quantify the number of bacteria, the phase‐contrast images, the images were converted into binary images with a white background using the default setting. The binary images were then analyzed using the Analyze Particles tool in Fiji‐ImageJ, in which the particles over 2 µm^2^ were classified as bacteria. To measure the number of bacteria in the high and low Hg^2+^ side, the number of particles in an area of 700 µm × 700 µm were measured, which included at t = 0 min at least 500 bacteria. To quantify the DiD intensity from BcSUVs on the bacteria, the binary image generated for the bacteria was used as a mask on the DiD image and subsequently, the mean DiD intensity within the mask was measured using Fiji ImageJ.

### 4.13 Statistical Analysis

All data were presented as the mean ± standard deviation. The statistical analyses were performed using Origin 2018 (OriginLab, USA). Comparisons between two groups were analyzed using a *t*‐test, and data from more than three groups were compared using a one‐way ANOVA.

## Conflicts of Interest

The authors declare no conflicts of interest.

## Supporting information




**Supporting File 1**: adma72950‐sup‐0001‐SuppMat.pdf.

**Supporting File 2**: adma72950‐sup‐0002‐MovieS1.avi.

**Supporting File 3**: adma72950‐sup‐0003‐MovieS2.avi.

**Supporting File 4**: adma72950‐sup‐0004‐MovieS3.avi.

## Data Availability

The data that support the findings of this study are available on request from the corresponding author.
