# Supplementary material for: Autonomous Cargo Transport with Biohybrid Microswimmers Enabled by Light‐Mediated Bacteria‐Cargo Communication
Source: Adv Mater. 2026 Mar 30;38(25):e72950. doi: 10.1002/adma.72950 (PMC13137770; doi:10.1002/adma.72950)
Supplement: Supplementary file 1 — Supporting File 1: adma72950‐sup‐0001‐SuppMat.pdf. [file ADMA-38-e72950-s002.pdf]

Supporting Information

Autonomous Cargo Transport with Biohybrid Microswimmers Enabled by Light-Mediated Bacteria-Cargo Communication

*Xiaoran Zheng, Yanjun Zheng, Ali Heidari, Seraphine Wegner**

Table S1. Buffer compositions used in this study.

Buffer Name	Composition
Buffer A	50 mM Tris, 300 mM NaCl, pH=7.4.
Wash buffer	50 mM Tris, 300 mM NaCl, 20 mM imidazole, pH=7.4.
Elution buffer	50 mM Tris, 300 mM $NaCl_2$, 500 mM imidazole, pH=7.4.
Working buffer	10 mM Tris, 150 mM NaCl, pH=7.4.
Motility buffer	10 mM K_3PO_4, 0.1 mM EDTA, 67 mM NaCl, pH=7, supplemented with 1% w/v glucose and 0.5% w/v BSA.

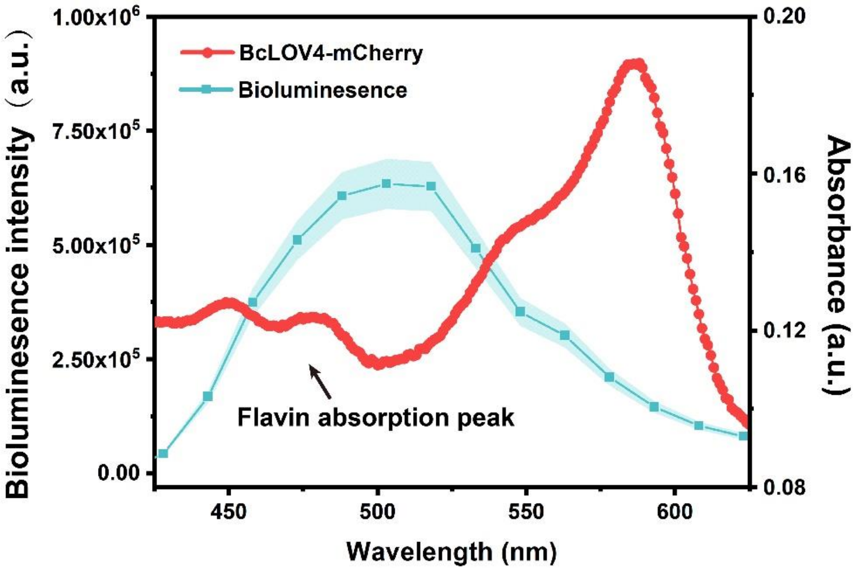

Figure S1. Bioluminescence spectrum of merR-lux bacteria after exposure to 1 μM Hg2+ for 1 hour overlayed with absorbance spectrum of purified BcLOV4-mCherry. The flavin absorption peak of BcLOV4-mCherry around 450-480 nm overlaps with the bioluminescence spectrum of merR-lux.

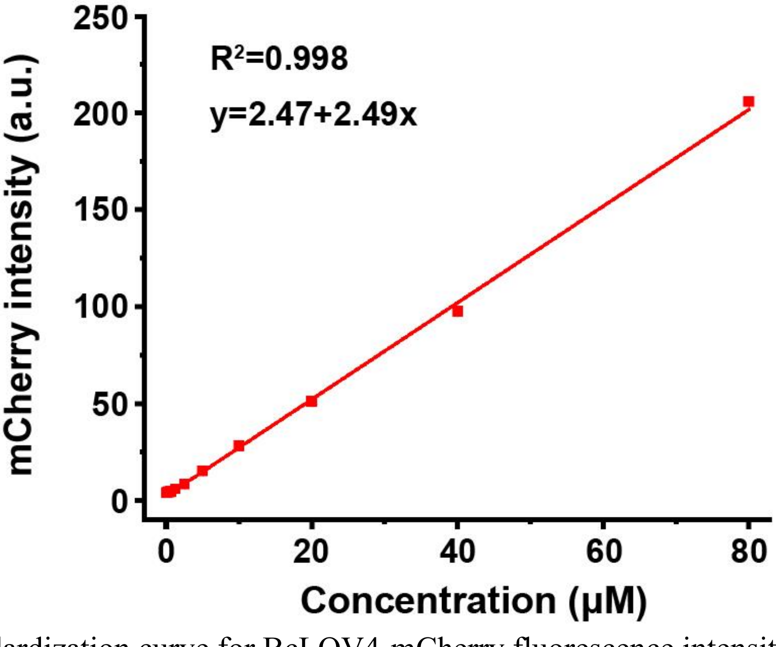

Figure S2. Standardization curve for BcLOV4-mCherry fluorescence intensity using the same CLSM imaging settings as for BcLOV4-mCherry recruitment to the bacteria.

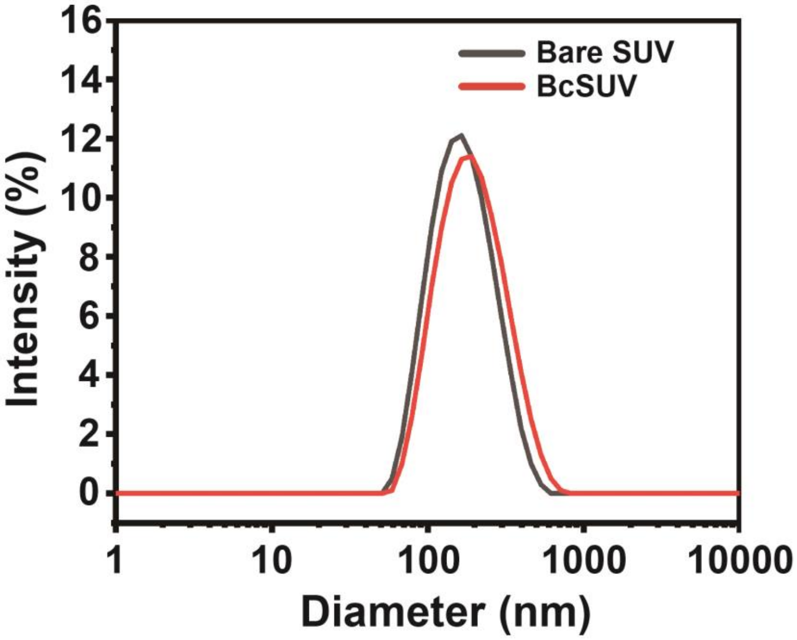

Figure S3. Size distribution of bare SUVs and BcSUVs were determined by dynamic light scattering.

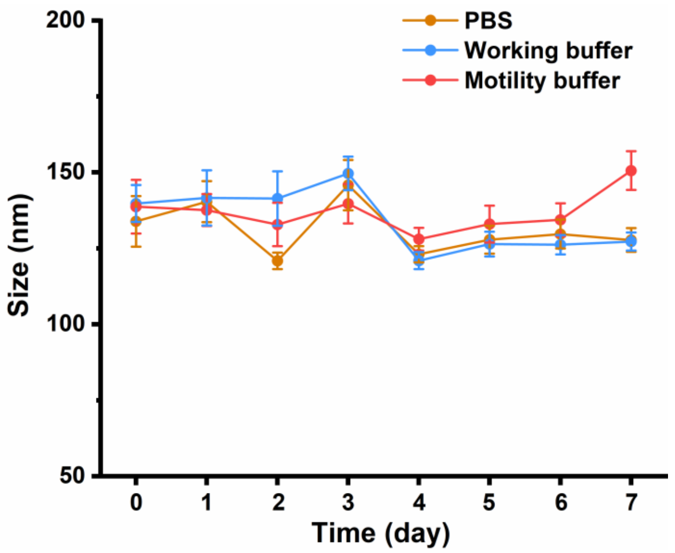

Figure S4. Size of BcSUVs keep in PBS buffer, working buffer and motility buffer for 7 days. Data were acquired from three independent samples.

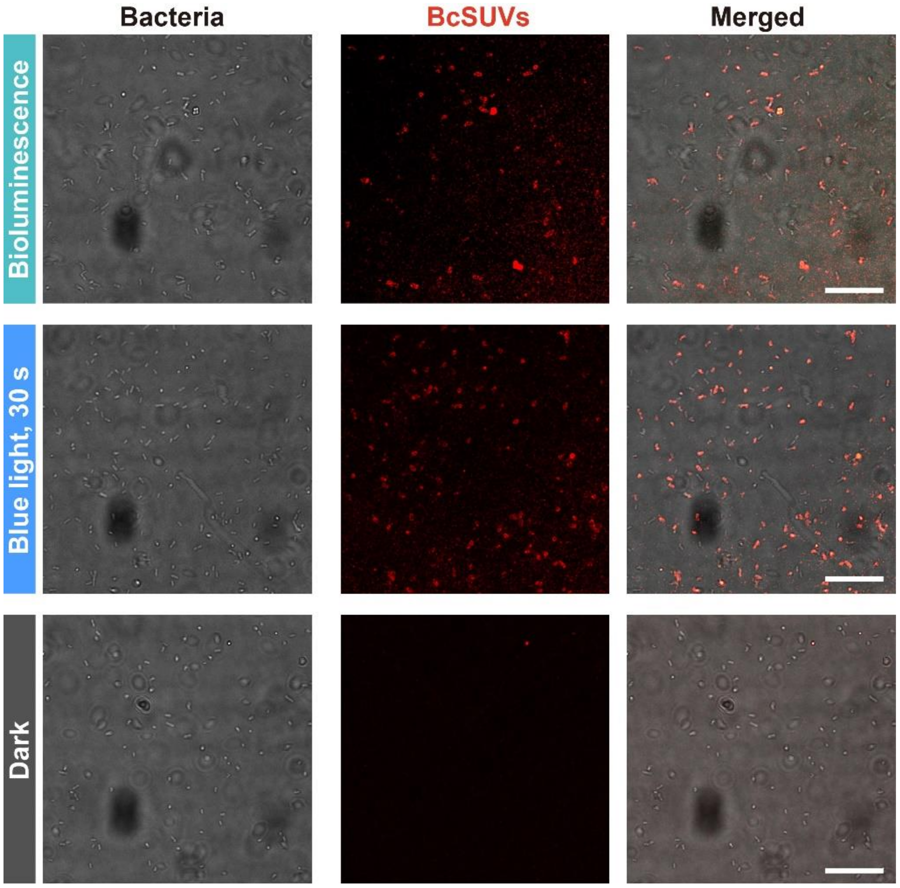

Figure S5. CLSM images of BcSUVs (DiD channel, red) interacting with merR-lux bacteria (bright field, gray) under bioluminescence (5 minutes), blue light (30 seconds) and in the dark (5 minutes). Scale bars are 20 µm.

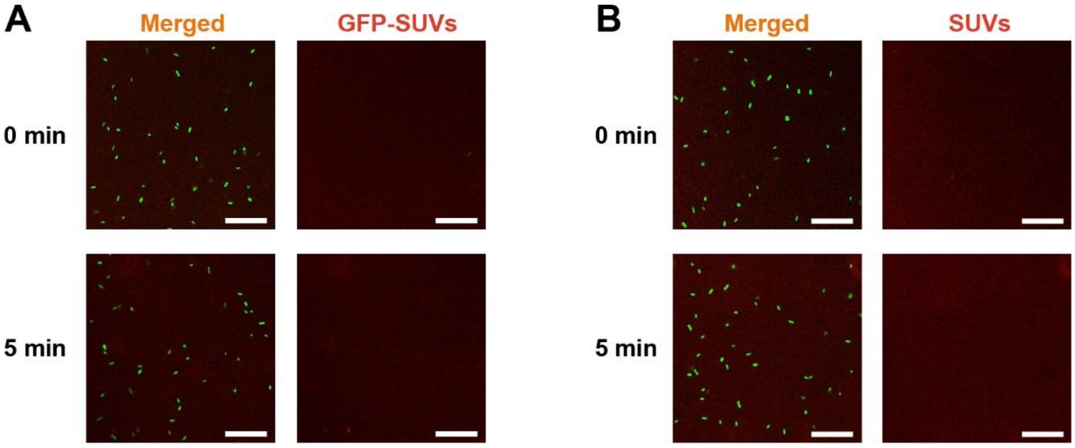

Figure S6. CLSM images of (A) non-functionalized and (B) GFP-functionalized SUVs (DiD, shown in red in the presence of bioluminescence producing merR-lux bacteria (mCherry, shown in green) after 5 minutes. Scale bars = 20 µm.

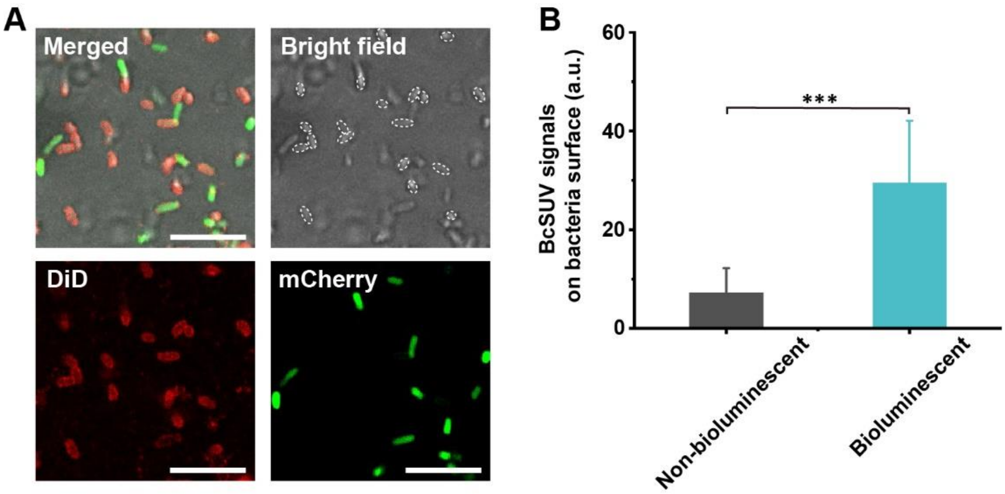

Figure S7. (A) CLSM images of BcSUVs (DiD, shown in red) incubated with cocultures of non-luminescent *mCherry* producing bacteria (mCherry, shown in green) and luminescent *merR-lux* bacteria. After 5-min incubation, BcSUVs only recruited to the membranes of bioluminescent *merR-lux* bacteria (unstained, dash line) but not to the surface of *mCherry* producing bacteria. (B) Quantification of binding efficiency of BcSUVs on non-bioluminescent and bioluminescent bacteria. Scale bars = 10 µm. Data were acquired from three independent experiments (n=3) with > 20 bacteria analyzed per experiment. $***p < 0.001$.

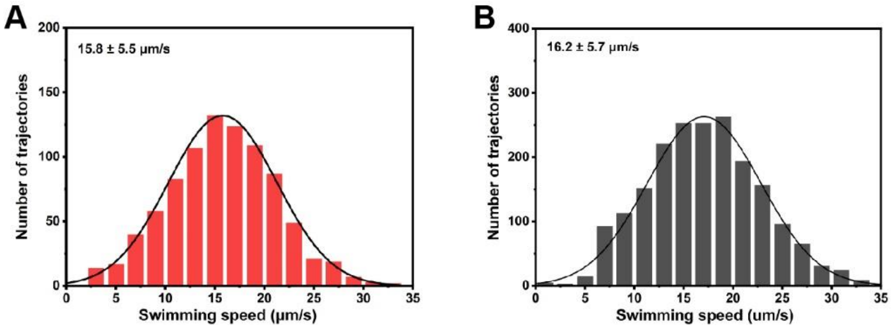

Figure S8. Swimming speed distribution of (A) *E. coli* MG1655 (VS202) in working buffer, and (B) *merR-lux E. coli* MG1655 (VS202) in working buffer that supplement 1000 nM Hg^{2+}. At least 500 trajectories were analyzed per experiment (n=2) and the number of trajectories was normalized against the total number of analyzed trajectories.

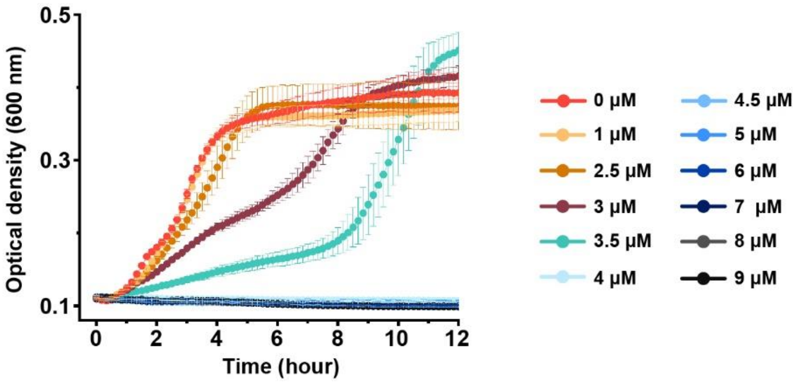

Figure S9. Optical density of merR-lux bacteria at 600 nm (OD_{600}) when exposed to different concentrations of Hg^{2+}.

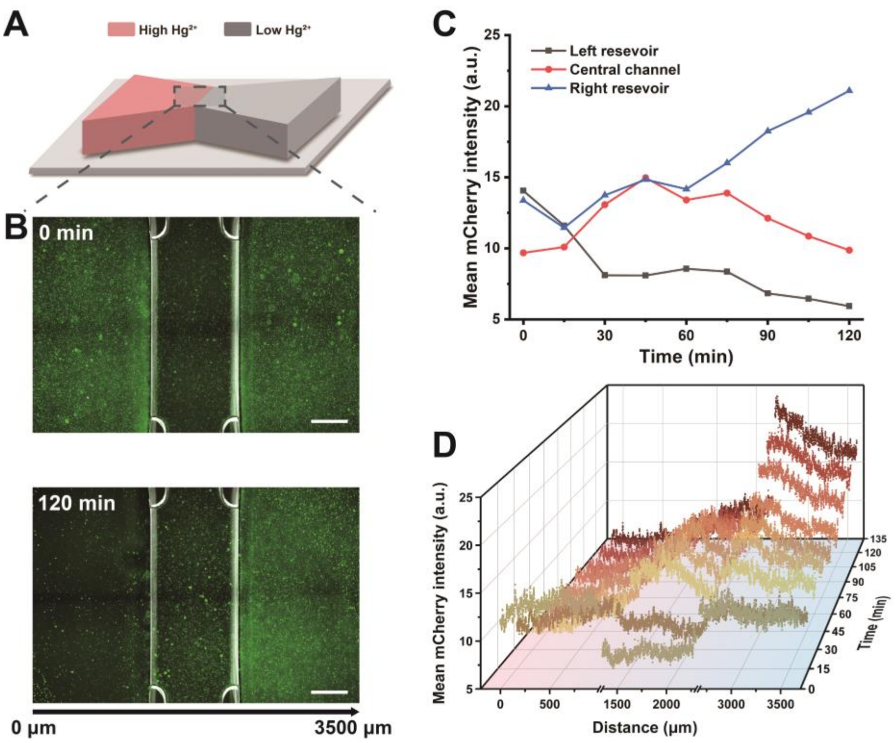

Figure S10. Chemotaxis behavior of merR-lux bacteria expressing mCherry. (A) Schematic of chemotaxis dish, high Hg^{2+} area is in red and low Hg^{2+} area is in grey. (B) Fluorescence microscopy images showing the distribution of merR-lux/mCherry bacteria (in green) before and after 120 minutes incubation. Scale bars are 400 μm. (C) Mean mCherry intensity of merR-lux/mCherry bacteria within the chemotaxis dish. (D) Change of mean mCherry intensity over time. The x-axis is defined by the line from the high Hg^{2+} to the low Hg^{2+} area.

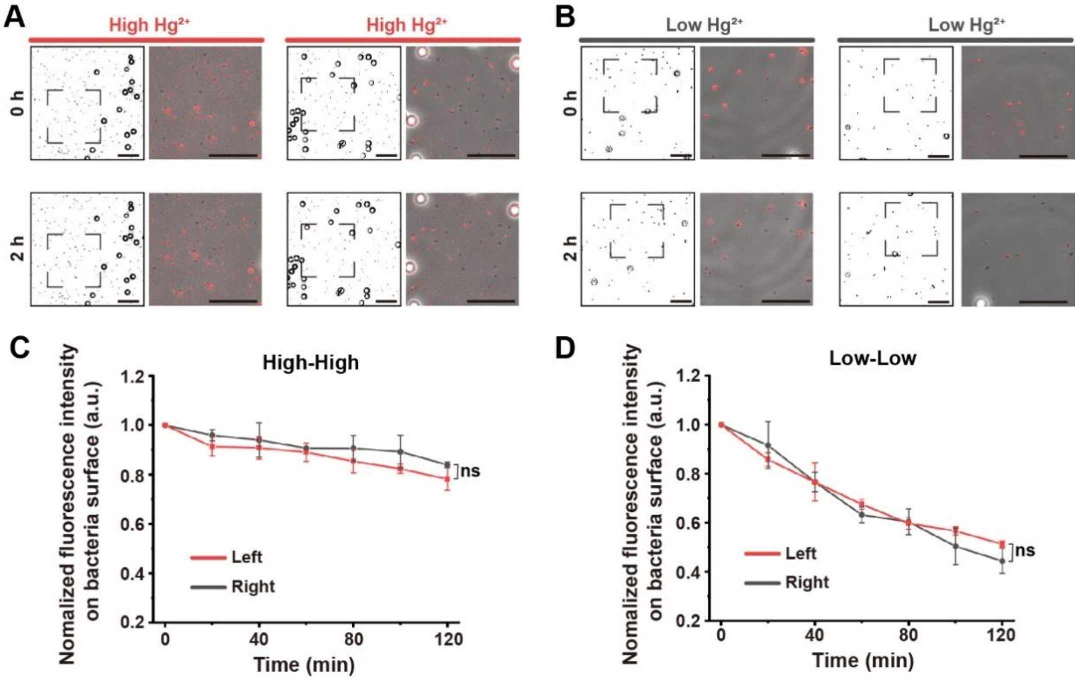

Figure S11. BcSUV signal on the bacterial surface in the chemotaxis chamber without a Hg^{2+} gradient. The distribution of merR-lux bacteria (shown as black dots) in the chemotaxis chamber with (A) equal high (1 µM) concentrations of Hg^{2+} on both sides, and (B) equal low (0 µM) concentrations of Hg^{2+} on both sides. Scale bars are 50 µm. (C) Change in BcSUV signal on the bacterial surface under equal high Hg^{2+} concentrations on both sides, and (D) change in BcSUV signal on the bacterial surface under equal low Hg^{2+} concentrations on both sides of the chemotaxis dish over time. ns: not significant.

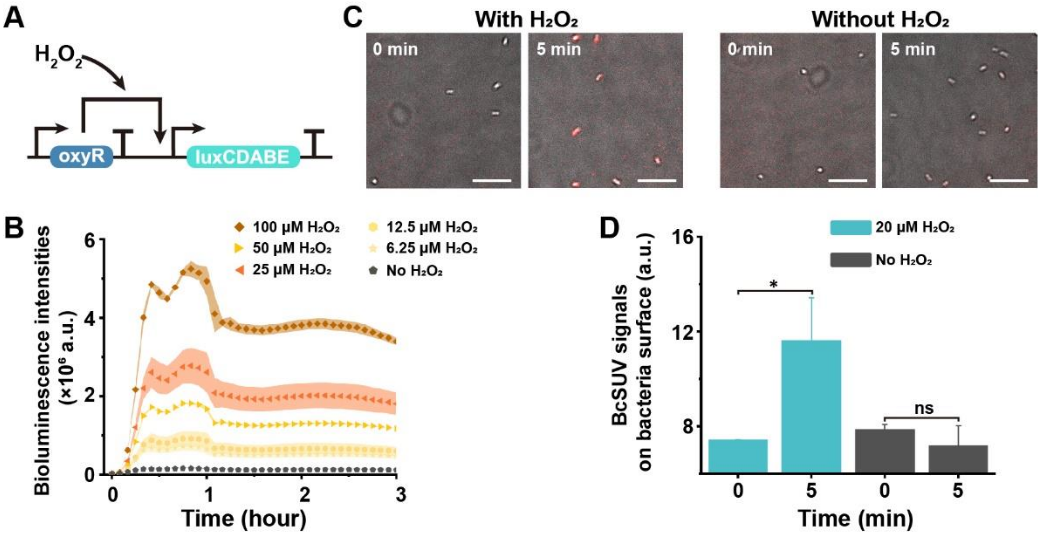

Figure S12. BcSUV recruitment induced by bacterial bioluminescence in response to H_2O_2. (A) Schematic of the sensor gene circuit (*oxyR-lux*) that responds to H_2O_2 with bioluminescence. In the presence of H_2O_2, the expression of the downstream luciferase cassette is activated in *E. coli*. (B) Bioluminescence signal from *oxyR-lux* transfected *E. coli* in the presence of different H_2O_2 concentrations. (C) CLSM images of BcSUV recruitment on the bacterial surface in the presence of 20 µM H_2O_2 or without H_2O_2. (D) Quantification of BcSUV signals on the bacterial surface under different H_2O_2 conditions. Scale = 10 µm. At least 10 bacteria were analyzed per experiment (n=2). *$p < 0.05$, ns: not significant.
